# Population Characteristics in Justice Health Research Based on PubMed Abstracts From 1963 to 2023: Text Mining Study

**DOI:** 10.2196/60878

**Published:** 2024-11-22

**Authors:** Wilson Lukmanjaya, Tony Butler, Patricia Taflan, Paul Simpson, Natasha Ginnivan, Iain Buchan, Goran Nenadic, George Karystianis

**Affiliations:** 1 School of Population Health University of New South Wales Sydney Australia; 2 Institute of Population Health Liverpool University Liverpool United Kingdom; 3 School of Computer Science University of Manchester Manchester United Kingdom

**Keywords:** epidemiology, PubMed, criminology, text mining, justice health, offending and incarcerated populations, population characteristics, open research, health research, text mining study, epidemiological criminology, public health, justice systems, bias, population, men, women, prison, prisoner, researcher

## Abstract

**Background:**

The field of epidemiological criminology (or justice health research) has emerged in the past decade, studying the intersection between the public health and justice systems. To ensure research efforts are focused and equitable, it is important to reflect on the outputs in this area and address knowledge gaps.

**Objective:**

This study aimed to examine the characteristics of populations researched in a large sample of published outputs and identify research gaps and biases.

**Methods:**

A rule-based, text mining method was applied to 34,481 PubMed abstracts published from 1963 to 2023 to identify 4 population characteristics (sex, age, offender type, and nationality).

**Results:**

We evaluated our method in a random sample of 100 PubMed abstracts. Microprecision was 94.3%, with microrecall at 85.9% and micro–*F*_1_-score at 89.9% across the 4 characteristics. Half (n=17,039, 49.4%) of the 34,481 abstracts did not have any characteristic mentions and only 1.3% (n=443) reported sex, age, offender type, and nationality. From the 5170 (14.9%) abstracts that reported age, 3581 (69.3%) mentioned young people (younger than 18 years) and 3037 (58.7%) mentioned adults. Since 1990, studies reporting female-only populations increased, and in 2023, these accounted for almost half (105/216, 48.6%) of the research outputs, as opposed to 33.3% (72/216) for male-only populations. Nordic countries (Sweden, Norway, Finland, and Denmark) had the highest number of abstracts proportional to their incarcerated populations. Offenders with mental illness were the most common group of interest (840/4814, 17.4%), with an increase from 1990 onward.

**Conclusions:**

Research reporting on female populations increased, surpassing that involving male individuals, despite female individuals representing 5% of the incarcerated population; this suggests that male prisoners are underresearched. Although calls have been made for the justice health area to focus more on young people, our results showed that among the abstracts reporting age, most mentioned a population aged <18 years, reflecting a rise of youth involvement in the youth justice system. Those convicted of sex offenses and crimes relating to children were not as researched as the existing literature suggests, with a focus instead on populations with mental illness, whose rates rose steadily in the last 30 years. After adjusting for the size of the incarcerated population, Nordic countries have conducted proportionately the most research. Our findings highlight that despite the presence of several research reporting guidelines, justice health abstracts still do not adequately describe the investigated populations. Our study offers new insights in the field of justice health with implications for promoting diversity in the selection of research participants.

## Introduction

Studies investigating the health needs of offender populations represent an emerging discipline called epidemiological criminology [1,2] and are affected by factors such as funding, complex and multilayered ethics approvals, access to prisoners or community-based offender populations, data quality, and reporting bias [3-6]. Understanding this population’s unique needs enables researchers and policy makers to target specific health and well-being needs rather than generalizing across all groups [7].

When researchers fail to accurately report their research, biases can occur [8]. For that reason, health research reporting has evolved with the introduction of STROBE (Strengthening the Reporting of Observational Studies in Epidemiology) [9], CONSORT (Consolidated Standards of Reporting Trials) [10], SPIRIT (Standard Protocol Items: Recommendations for Interventional Trials) [11], and PRISMA (Preferred Reporting Items for Systematic Reviews and Meta-Analyses) [12] statements, which provide guidelines and templates for investigators to structurally report their findings in concise yet detailed manners.

Developing effective population prevention and intervention strategies requires evidence-based reporting of the studied population [13]. A 2018 synthesis of reviews on global prisoner health concluded that detained adolescents were not the focus of any of the included studies despite evidence of health inequalities within that particular population [14]. Furthermore, minority groups exhibit varying morbidity and mortality rates, suggesting distinct health risks and outcomes [15]. Establishing conclusions generated from a minority population toward larger ones, and vice versa, has the potential to lead to ineffective interventions [16,17]. Therefore, it is imperative to accurately report the characteristics of populations involved in research to ensure the transparency and reproducibility of related studies.

As more scientific literature becomes available, the task of manually reading, extracting, and synthesizing knowledge from large numbers of epidemiological studies becomes more time-consuming [18-20]. Automated applications offer investigators the opportunity to quickly and efficiently detect, summarize, and incorporate key information from relevant literature [21,22]. However, few studies have attempted to determine a whole-of-discipline perspective by examining the scope and quality of peer-reviewed outputs over time. Previous efforts have shown that it is possible to automatically identify information from PubMed abstracts of published studies [19,20,23-33]. Most research has focused on extracting specific study information (eg, study design, populations, country, effect size, outcomes, confounding factors, and intervention) from PubMed abstracts that are relevant to an entire discipline such as justice health [20,31,33] and biomedicine [23,28,29], summarizing the findings of clinical trials [24-27,32], or consolidating detail findings across a particular topic (eg, obesity [19] and environmental studies [30]) using several text mining approaches that range from rule-based methods to machine learning with varying degrees of success.

Health research, including that related to the justice system, is indexed in bibliographical databases that publish the abstracts of such studies. Abstracts are written in a relatively structured format following each journal’s reporting style and aim to improve communication. They are publicly available in digital form and not behind a paywall, enabling easy large-scale research. The largest database is PubMed, developed by the National Institutes of Health’s National Library of Medicine, which provides access to millions of citations from biomedical journals [34]. For example, PubMed has more than 34,000 published articles in the justice health area alone [33].

Epidemiology is a field with its own dictionary with related studies describing characteristics of participants; implemented study designs; and associations between exposures, risk factors, and outcomes adhering to a semistructured reporting style [19,30,35]. For this reason, we hypothesized that a simple text mining (ie, rule-based) approach (ie, syntactical rules that can identify characteristics of interest) could provide a quicker and more effective means to extract key information from the whole discipline of justice health as opposed to the application of more advanced machine learning methods that would require a large number of annotated training data or black-box algorithms that may carry an increased risk of potential biases [36,37]. In this study, we applied a rule-based method on 34,481 PubMed justice health abstracts from 1963 to 2023 to automatically extract a set of population characteristics (age, sex, nationality, and offender type) and highlight whether there are biases or gaps in this area from a participant perspective.

## Methods

### Data

We conducted a literature search in PubMed using an expanded version of an existing query [20] containing search terms related to offenders and prisons. These were combined with either the Medical Subject Headings term “epidemiology” to capture all types of epidemiological studies or with all the available (in PubMed) publication types (eg, meta-analysis and clinical trial) to ensure the results will return clinical trials and secondary research (eg, review). We also added terms related to randomization/natural experiments and synthetic control. These choices prevented articles that made only passing references to prisoner and offender studies from entering the dataset, resulting in a high-quality corpus for our analysis. The search was restricted to English-language articles that have an abstract and involved only human participants (ie, veterinary research was excluded). The full query (Textbox 1) was run on July 20, 2023.

**Search query**

“prison OR borstal OR jail OR jails OR gaol OR gaols OR penitentiary OR custody OR custodial OR (corrective AND (service or services)) OR ((correctional or detention) AND (centre or centres OR center OR centers OR complex OR complexes or facility or facilities)) OR (closed AND (setting)) OR prisoner OR prisoners OR incarcerated OR criminals OR criminal OR felon OR felons OR remandee OR remandees OR delinquent OR delinquents OR detainee OR detainees OR convict OR convicts OR cellmate OR cellmates OR offenders OR offender OR ((young OR adolescent) AND (offender OR offenders)) OR ((delinquent OR incarcerated) AND youth) OR (juvenile AND (delinquents OR delinquent OR delinquency OR detainee OR detainees OR offender OR offenders)) OR ((young) AND (people) AND (in) AND (custody)) OR ((justice) AND (involved) AND (youth)) OR ((incarcerated) AND (young) AND (people OR person OR persons)) OR ((juvenile OR juveniles) AND (in) AND (custody)) AND english [lang] AND (“epidemiology”[Subheading] OR “epidemiology”[MeSH Terms] OR epidemiology[Text Word] OR clinical study[publication type] OR case reports[publication type] OR clinical trial[publication type] OR clinical trial, phase i[publication type] OR clinical trial, phase ii[publication type] OR clinical trial, phase iii[publication type] OR clinical trial, phase iv[publication type] OR comparative study[publication type] OR controlled clinical trial[publication type] OR evaluation study[publication type] OR meta-analysis[publication type] OR multicenter study[publication type] OR observational study[publication type] OR pragmatic clinical trial[publication type] OR randomized controlled trial[publication type] OR review[publication type] OR systematic review[publication type] OR twin study[publication type] OR validation study[publication type] OR non randomized trial[text word] OR non randomised trial[text word] OR randomization experiment OR randomisation experiment OR natural experiment OR synthetic control)”.

### Text Mining

#### Dictionaries

To identify the reported sex, we used various indicators (eg, boys, girls, men, women, males, females, transgender, and trans). A total of 26 terms were used (Multimedia Appendix 1). We also compiled a list of offenses [38] including common synonyms (eg, “sex crime,” “sex offending,” and “sexual offending”), acronyms (eg, “ADVO [apprehended domestic violence order]”), and descriptive sentences (eg, “breach of parole” and “assault with intent to commit rape”). We also included grammatical variations of these offenses to expand the scope of our dictionary. A total of 1036 terms were used.

For nationalities, we used 3 dictionaries that indicate a place of origin: one for overall nationalities (n=1575), one for country names (n=363), and one for the largest cities of the world (n=317). We included nationalities (eg, Czechoslovakian) and countries (eg, Yugoslavia) that no longer exist as well as variations of the same nationality and country/region (eg, “Dominicans,” “Dominicanes,” “United Kingdom,” “Great Britain,” “Britain,” and “UK”) [39]. Considering how our previous research on examining first author affiliations from justice health PubMed abstracts demonstrated that the United States was the number one country in sheer publication outputs in this area [20], we added 3 more dictionaries for the US states (n=50), counties (n=3135) and the largest US cities (n=200).

We also used a dictionary of 259 commonly used terms to describe offending and incarcerated populations (eg, criminals, incarcerated, reoffending, juvenile, and delinquent; Multimedia Appendix 2).

#### Rule-Based, Text Mining Approach

From our query results, we randomly selected 100 abstracts as our training set. The training set was manually and independently annotated by 2 authors with epidemiological and public health backgrounds (GK and TB) for the 4 population characteristics (ie, nationality, age, sex, and offender type) based on specific annotation guidelines. The returned annotator agreement, which was calculated as the absolute agreement rate [40] at the abstract level for all 4 characteristics, was 92%, indicating very good annotation consistency. Cases of disagreement were reviewed and were viewed as incorrect omissions rather than instances where the annotators were highlighting a completely different characteristic mention. Following this, the annotations of the training set were rectified with the agreement of both annotators.

We developed rules based on common lexical patterns observed in the training set that suggest the presence of any of the 4 characteristics. The lexical patterns use frozen syntactical expressions as anchors for certain elements built through verbs, noun phrases, prepositions, and semantic placeholders that can be identified by the dictionary application as indicating a characteristic. For example, the sentence “characteristics of sex offenders in” mentions the offender type as “sex offenders.” To identify this, the semifrozen lexical expressions “characteristics of” and “in” are matched via 2 regular expressions, and “sex offenders” gets a match through the offender-type dictionary. More than 1 lexical pattern may be matched in an abstract referring to 1 or more mentions of a characteristic (which can be duplicates).

An additional sample of 100 randomly selected abstracts was used to serve as the development set in order to optimize the performance of our method by (1) refining and attempting to generalize our rules (in order to avoid instances of overfitting, ie, rules that worked efficiently only in lexical patterns encountered in the training set) and (2) by increasing the scope of our manual engineering dictionaries by adding extra terms that might have not been encountered in the training set. A total of 140 rules were crafted: 11 for nationality, 47 for age, 4 for sex, and 78 for offender type (Multimedia Appendix 3 shows rule examples for each characteristic). To convert the observed lexical patterns into rules, we used General Architecture for Text Engineering (GATE), a text mining framework and its Java Annotations Pattern Engine, a pattern matching language for GATE [41]. GATE was selected because it enables the support of rule-based, text mining approaches and has an effective graphical user interface.

#### Data Standardization

To enable descriptive analysis of the extracted results, mentions of age, sex, nationality, and offender type were standardized by using a simple Python script. Unique values from each characteristic were manually inspected by 3 authors (GK, PS, and TB) to identify synonyms (eg, sex offenders, sex offending, and sexual offenders), which then were assigned a respective term (eg, sex offender). For age, 2 types of mentions were identified: numeric (eg, 18 to 24 years old) and textual (eg, adolescents and adults). We categorized the numerical values according to the Australian Bureau of Statistics’ 7 age groups: younger than 18 (minors), 18-24, 25-34, 35-44, 45-54, 55-64, and 65+ years old [42-46]. We also assigned a numeric range for the textual mentions (Table 1). If “adults” mentions were stated, this was placed into the “unknown adult” category.

**Table 1:** Classification of standardized age textual mentions from PubMed abstracts.

Term	Age range (years)	Classification
Children, minors, juveniles, delinquents, school children, boys, girls	<18	Minors
Juvenile, delinquent	10-17	Minors
Adolescent, teen	13-19	Minors 18-24 years old
Young, youths, young offenders	15-24	Minors 18-24 years old
Adults	18+	Unknown adults

To standardize sex mentions, 5 categories were used: male, female, transgender individuals (ie, mentions of transgender individuals without specification), transgender men, and transgender women. For nationality mentions, those that belonged to US counties, cities, and states were standardized as “American,” whereas nationalities that are not in use anymore (eg, “Czechoslovakian”) were assigned a miscellaneous status.

For the offender type, due to the different levels of information that each mention might bear (eg, serial rapist), we used a more generic (when possible) grouping. For example, for populations involved with rape, we maintain the specific offense as rape and assign a higher offense node as “sex offence.” A total of 6 categories were created (child crime–related [including child sex abuse] offender, sex offender, violent offender, nonviolent offender, mentally ill offender, and drug-related offender). We also created an additional category called “miscellaneous” to include other nonspecific descriptions (eg, “high risk offenders,” “ex-offenders,” “juveniles,” and “delinquents”) that could not be mapped to any of the other categories (Multimedia Appendix 4). To obtain results at the abstract level for each abstract, we eliminated any duplicate mentions of the standardized characteristics. Table 2 shows some examples of standardizing extracted population mentions according to the 4 defined characteristics.

**Table 2:** Examples of standardized extracted mentions of the 4 population characteristics (ie, age, sex, nationality, and offender type) including attributes that describe offender types.

Extracted mention	Characteristic	Standardized version	Offender type
Women	Sex	Female	—^a^
Boys and girls	Sex	Male and female	—
Women with borderline personality disorder	Sex and offender type	Female and mentally ill offender	Borderline personality disorder
Child molester	Offender type	Child crime–related offender	Child sex abuse
Male sex offenders	Offender type	Sex offender	—
Serial rapists	Offender type	Sex offender	Rape
Psychotic inmates	Offender type	Mentally ill offender	Psychosis
Ex-offenders	Offender type	Miscellaneous	Ex-offender
Age 18-25 years	Age	18-24 years and 25-34 years	—
Iowa	Nationality	American	—
Norway	Nationality	Norwegian	—

^a^Not applicable.

## Results

### Text Mining Evaluation

The system’s performance was evaluated at the abstract level and used the standard definitions of the precision, recall, and *F*_1_-score metrics [47]. True positive (TP) was defined as the identification of either all the correct mentions of a population characteristic or the extraction of a number of mentions for one population characteristic, even if the system failed to pick up some mentions in an abstract. For example, if an abstract had 2 mentions of the female sex (eg, “females” and “women”), then the detection of either one or both mentions would be considered a TP at the abstract level with “female” being the standardized sex in this abstract. The same process was applied in cases where there can be more than one different mention of a population characteristic (eg, mentions of 2 different nationalities for the investigated population). A false positive (FP) is an identification of an incorrect mention of a population characteristic while a false negative (FN) is an incorrectly ignored mention of a population characteristic. Precision measures the accuracy of TP predictions, recall measures the completeness of identifying TPs, and the *F*_1_-score balances both by combining them in a single metric.

Overall, at the abstract level, the mean precision and recall were 95.2% and 90.9%, respectively, whereas the *F*_1_-score was 93%. However, since the number of mentions between the 4 characteristics varied drastically in the evaluation set, we reported on the micro values of precision, recall, and *F*_1_-score to offer a more weighted approach to the system’s performance. Microprecision was 94.3%, with microrecall at 85.9% and micro–*F*_1_-score at 89.9%. The largest recall drop was observed in age (6.2%), and it was the only recall with a value below 80% (78.8%), while nationality had the highest recall (95.2%). Sex had the highest precision (100%), followed by age (97.6%; Table 3). The highest *F*_1_-score was observed for sex (96.4%), followed by nationality (93%). Age and offense type had similar *F*_1_-scores with 87.1% and 87.3%, respectively (Table 3).

**Table 3:** Precision, recall, and F1-score for the training, development, and evaluation sets, including the number of true positives (TPs), false positives (FPs), and false negatives (FNs) at the abstract level for age, sex, offender type, and nationality.

Characteristics and dataset				TP		FP		FN		Precision (%)		Recall (%)		*F*_1_-score (%)
**Age**														
		Training		51		6		10		89.4		83.6		86.4
		Development		57		1		10		98.2		85.0		91.1
		Evaluation		47		1		11		97.6		78.8		87.1
**Sex**														
	Training		52		5		5		91.2		91.2		91.2	
	Development		54		6		5		90.0		91.5		90.7	
	Evaluation		54		0		4		100.0		93.1		96.4	
**Offender type**														
	Training		78		15		15		83.8		83.8		83.8	
	Development		94		11		8		89.5		92.1		90.7	
	Evaluation		98		8		19		92.4		83.7		87.3	
**Nationality**														
	Training		36		3		3		92.3		92.3		92.3	
	Development		32		5		1		86.4		96.9		92.3	
	Evaluation		40		4		2		90.9		95.2		93.0	

### Query Results

Our query returned 34,481 justice health study abstracts with the earliest recorded in 1963 (Multimedia Appendix 5). Half of the abstracts (17,039/34,481, 49.4%) did not have any characteristic mentions. Most abstracts either mentioned only age and nationality (1676/34,481, 4.9%) or age and offender type (1082/34,481, 3.1%). Only 1.3% (443/34,481) of abstracts reported all 4 characteristics (sex, age, offender type, and nationality).

### Age

A total of 5170 (14.9%) abstracts out of 34,481 reported the population’s age; of the 5170 abstracts, 3581 (69.3%) mentioned minors, 3037 (58.7%) mentioned adult populations, and 181 (3.5%) not specifying the age. The largest adult group was that of 18-24 years old (33.4%) followed by 35-44 years old (10.4%). Studies involving 55-64-year-olds had the lowest number of mentions (7.4%; Table 4).

**Table 4:** Number of justice health abstracts (n=5170) in PubMed from 1963 to 2023 reporting age. Note that 1 abstract can include more than 1 age group.

Initial age group		Abstracts, n (%)
Minors (<18 years)^a^		3581 (69.3)
**Adults (>18 years)** ^b^		
	18-24	1728 (33.4)
	25-34	519 (10.0)
	35-44	538 (10.4)
	45-54	398 (7.7)
	55-64	382 (7.4)
	65+	473 (9.1)
	Unknown adult	183 (3.5)

^a^Number of abstracts is 3581 (69.3%) out of 5170.

^b^Number of abstracts is 3037 (58.7%) out of 5170.

### Sex

**Figure 1:**
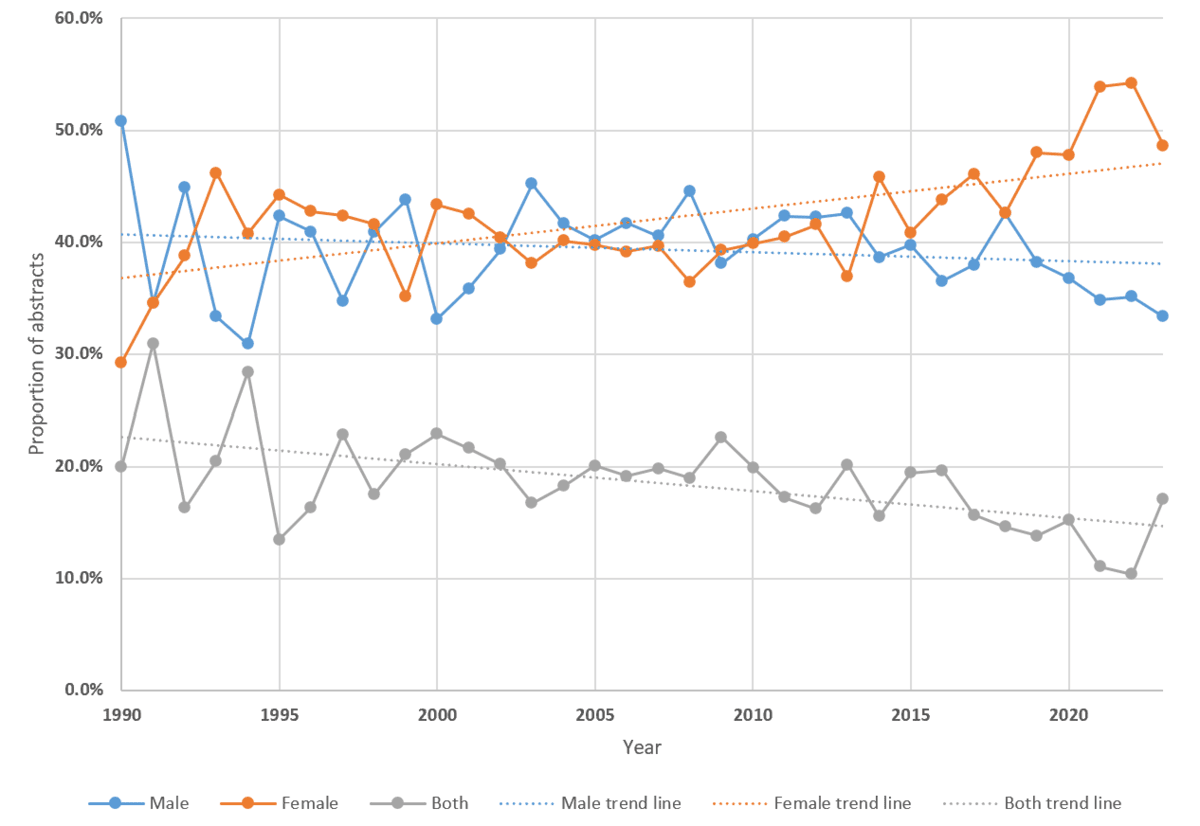
Proportions of PubMed study abstracts that reported female-only and male-only populations from 1990 to 2023. Due to the very low rates for transgender populations, these were not included in the graph.

A total of 8169 (23.7%) out of 34,481 abstracts reported the sex of the investigated population in the abstract. Around 39.7% (3241/8169) of the abstracts reported only male populations, 42.9% (3501/8169) reported only female populations, and 17.4% (1418/8169) reported both male and female populations. Less than 1% (n=21) of abstracts reported transgender populations. Although there has been a gradual increase in study rates involving only female populations since 1990 (see the trend line in Figure 1), from 2014 onward, a decrease was noted with regard to abstracts reporting only male populations, surpassed by abstracts reporting only female populations. In 2023, a total of 48.6% (105/216) of abstracts reported only female populations versus 33.3% (72/216) reporting only male populations (Figure 1; Multimedia Appendix 6 shows the rates per year in detail).

### Nationality

A total of 9525 (27.6%) out of 34,481 abstracts reported the nationality of the investigated population. The most common nationality was the United States (ie, American; 2992/9525, 31.4%), followed by the United Kingdom (786/9525, 9.2%) and Australia (730/9525, 7.6%; Table 5). However, to account for the size of the country’s population, which we assumed to be broadly linked to the size of its prisoner population (Pearson *r*=0.73), and this in turn being a potential driver of the volume of research reflected by the number of publications, we derived a publication rate based on the average prisoner population size over the period of 2000 to 2020 [48] and calculated a rate per 1000 prisoner population. In this case, the Nordic countries were in the top 4 in terms of publication rate followed by Australia (Table 5). Only 4 countries from Asia (China, India, Japan, and South Korea) and 1 country from Africa (South Africa) were in the top 20 of both crude and rate ranks.

**Table 5:** Top 20 most common nationalities reported in 9525 justice health articles in PubMed from 1963 to 2023, along with the respective continent, number of articles, prisoner population (average prisoner population 2000 to 2020), article rate per 1000 prisoners, and rate rank.

Crude rank	Country	Continent	Articles, n (%)	Prisoner population	Article rate per 1000 prisoners	Rate rank
1	United States	North America	2922 (31.41)	2,120,277	1.4	16
2	United Kingdom	Europe	993 (10.43)	88,274	11.2	9
3	Australia	Oceania	730 (7.66)	30,685	23.8	5
4	Canada	North America	498 (5.23)	38,321	13.0	8
5	China	Asia	371 (3.90)	1,627,290	0.2	20
6	Germany	Europe	340 (3.57)	68,437	5.0	10
7	France	Europe	281 (2.95)	62,158	4.5	11
8	Sweden	Europe	261 (2.74)	6510	40.1	3
9	India	Asia	249 (2.61)	385,832	0.6	18
10	Netherlands	Europe	249 (2.61)	14,470	17.2	7
11	Italy	Europe	227 (2.38)	56,090	4.0	12
12	Japan	Asia	202 (2.12)	65,348	3.1	13/14
13	Spain	Europe	193 (2.03)	61,751	3.1	
14	Brazil	South America	162 (1.70)	509,602	0.3	19
15	South Africa	Africa	146 (1.53)	164,629	0.9	17
16	Norway	Europe	138 (1.45)	3289	42.0	1
17	Switzerland	Europe	135 (1.42)	6257	21.6	6
18	Finland	Europe	135 (1.42)	3238	41.7	2
19	Denmark	Europe	125 (1.31)	3729	33.5	4
20	South Korea	Asia	94 (0.99)	52,989	1.8	15

### Offender Type

A total of 4814 (13.9%) out of 34,481 abstracts mentioned the offender type. Offenders with mental illness were reported in 17.4% (840/4814) of the PubMed abstracts, followed by sex offenders (620/4814, 12.9%). Child crime–related offenders (eg, child abusers) had the lowest number of mentions with 1.7% (84/4814; Table 6).

**Table 6:** Number of justice health abstracts (n=4814) in PubMed with an offender type across female and male populations. Note that 1 abstract might have more than 1 offender type and might include both male and female populations.

Offender type	Frequency, n (%)	Male, n (%)	Female, n (%)	Unknown sex, n (%)
Miscellaneous	3389 (70.4)	1162 (34.3)	942 (27.8)	1741 (51.4)
Mentally ill offender	840 (17.4)	193 (23.0)	103 (12.3)	596 (71.0)
Sex offender	620 (12.9)	211 (34.0)	81 (13.1)	386 (62.3)
Drug-related offender	521 (10.8)	111 (21.3)	84 (16.1)	356 (68.3)
Violent offender	364 (7.6)	134 (36.8)	77 (21.2)	201 (55.2)
Nonviolent offender	96 (2.0)	25 (26.0)	16 (16.7)	67 (69.8)
Child crime–related offender	84 (1.7)	26 (31.0)	16 (19.0)	54 (64.3)

**Figure 2:**
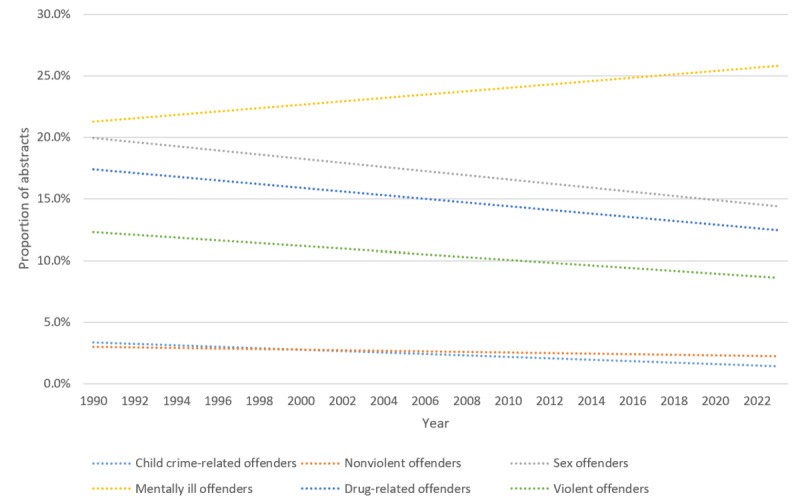
Trend lines for the rates of the 6 offender types in justice health abstracts (n=4814) in PubMed from 1990 to 2023.

From 1990 to 2023, the overall number of PubMed abstracts with an offender type increased (Multimedia Appendix 7). However, the rate (ie, the number of PubMed abstracts with a specific offender type divided by the total number of PubMed abstracts that had a mention of an offender type) revealed a general increase for offenders with mental illness. Mentions for sex, drug-related, nonviolent, and violent offenders had an overall decrease, with the biggest noted for sex offenders (10%; Figure 2).

**Figure 3:**
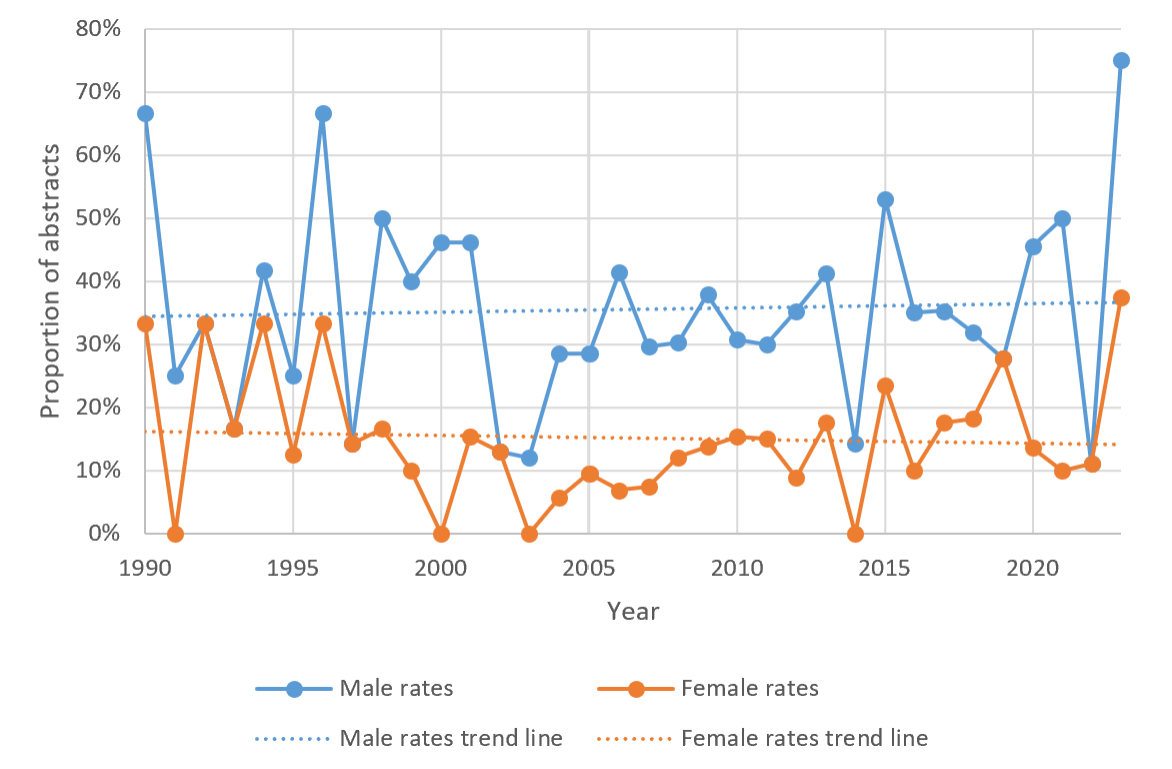
Male and female sex offender rates in justice health abstracts (n=4814) in PubMed from 1990 to 2023.

When the sex offenders are broken down by male and female, the rates of study abstracts through time remain roughly the same since 1990, with an average rate of 33% for male offenders and 15.5% for female offenders. A minimal increase in male sex offenders and a minimal decrease in female sex offenders were observed (Figure 3).

## Discussion

### Principal Findings

This text mining study demonstrated that key population characteristics (age, sex, offending type, and nationality) can be derived by applying text mining to a large corpus of study abstracts available in PubMed. Our findings enable researchers to investigate the presence of potential research and knowledge gaps over time that arise from examining certain offending groups within an entire discipline. Half of the abstracts (17,039/34,481, 49.4%) did not report any characteristics, while the number of abstracts that mentioned 1 characteristic ranged from 13.9% (n=4814; offender type) to 27.6% (n=9525; nationality). This highlights a larger problem regarding the reporting of the necessary information in abstracts for the description of populations within the justice health area as only 1.3% (n=443) of our sample abstracts reported sex, age, offender type, and nationality.

Previous research has showcased that despite several reporting guidelines covering observational, experimental, and secondary study reporting (eg, STROBE, CONSORT, SPIRIT, and PRISMA) [9-12], justice health abstracts do not appear to adequately detail their study designs and examined variables [31,34] and, based on our results, nor do they adequately describe the population under investigation. The importance of population description in research is needed to not only understand predictors for recidivism but also to enable the conduction of meta-analyses and other future studies [49].

### Age

We initially standardized the extracted age into 2 groups (younger than 18 years and older than 18 years) to examine age-related trends in offending populations. However, age was mentioned in 14.9% (5170/34,481) of our PubMed abstract sample; thus, this finding should be taken with caution. Although it has been suggested that the justice health area should focus more on young people [14], our results showed that 69.3% (3581/5170) of abstracts mentioned a population aged <18 years. This finding reflects the rise in youth involvement in the youth justice system [50]: the United States saw a 30% increase in juvenile cases between 1985 and 2009 [51], and Australia noted a recent increase of 6% from 2021-2022 to 2022-2023 [52].

The high number of abstracts reporting minors in the justice health area could also be explained through reporting practices. It is possible that the majority of researchers who investigate minor populations are more likely to specify their age. Most abstracts referred to the investigated population in generic terms such as “offenders” or “incarcerated individuals,” which could imply adult populations. This would separate them from younger people who are described by more specific terms such as “adolescent,” “juvenile,” and “delinquent.” This, however, was not taken into account for this research. Considering this and along with the inspection of full-text studies that might describe in detail the age of the participant population, our finding could be different.

### Sex

Although male individuals make up the overwhelming majority of incarcerated populations (10.9 million worldwide vs <1 million for female individual), there was an overrepresentation of studies involving female offenders, suggesting that male individuals in prison are an underresearched group [53]. Increased research into female populations since 2000 aligns with an increase in the number of incarcerated female individuals worldwide since 2000 [54], with female offender studies rising from 29.2% (19/65) in 1990 to 48.6% (105/216) in 2023. Conversely, male-focused research decreased from 50.8% (33/65) in 1990 to 33.3% (72/216) in 2023. This disparity evokes a consideration of equity in justice health research. Equity and not equality should be prioritized in health [55,56]. Therefore, it is possible that although inequality is shown through rates of research between male and female offending populations, an equity approach can contribute to our understanding of why there is disproportionately more research on female populations.

However, since only 23.7% (8169/34,481) of our data sample reported sex, it is possible that the remaining studies that did not detail the population’s sex in the abstract focused on male populations. Given that most prisons hold male prisoners only, investigators focusing on female populations in the justice health area might be better at reporting female sex. Nevertheless, this highlights the need for more detailed reporting in PubMed abstracts, to allow other researchers to accurately synthesize information more effectively and accurately.

### Nationality

Nationality was the population characteristic with the highest prevalence in our sample (9525/34,481, 27.6%). Using the crude rank, the United States was the most common nationality. However, by implementing the publication rate, the United States dropped to the 19th place (ie, among the most common nationalities), with the Nordic countries (Norway, Finland, Sweden, Denmark) occupying the top 4 spots and Australia the fifth one. Previous research analyzing PubMed justice health abstracts showed similar rankings for the Nordic countries in terms of their total published outputs [20]. Nordic countries are regarded as having a progressive approach to offender rehabilitation, with proportionally lower numbers of incarcerated individuals and recidivism rates compared with many other countries [57,58]. However, these results are based only on 27.6% of PubMed abstracts with a reported nationality; so, it is possible that in full-text studies, the actual nationality of the examined population is described, which could in hindsight reveal a different picture in the rankings.

### Offender Type

Offenders with mental illness were the most common group identified from the abstracts (840/4814, 17.4%). In the United States, it has been estimated that 24% of the inmate population have a mental illness [59], with approximately 50% to 75% of the 2 million young people meeting the criteria for a mental health disorder [60,61]. In the last 10 years, reliance on the juvenile justice system to meet its population’s mental health needs has increased and so has the research to examine the effectiveness of intervention and treatment programs [62]. Offenders with mental illness have higher rates of recidivism, exhibiting rehabilitation needs and prison adjustment difficulties that differentiate them from the general offender population [63,64]. Our results highlight the depth of this problem with researchers examining a total of 58 unique mental illness concepts (Multimedia Appendix 8) in the last 70 years, ranging from behavioral disorders (eg, attention-deficit/hyperactivity disorder) to mood disorders (eg, depression and bipolar disorder) and anxiety disorders (eg, posttraumatic stress disorder), with substance use disorders and intellectual disability receiving the most focus.

Most research on the longitudinal pattern of criminal careers has focused on generally violent offenders [65], which could explain the relatively low number of abstracts mentioning nonviolent offenders (96/4814, 2%) involved, for example, with theft and shoplifting. This indicates the need to investigate a more diverse range of offender groups [66,67]. Crimes such as theft, stalking, and driving under the influence may cause significant harm toward others, and yet, there is a lack of related work focusing on cases of, for example, fraud and sextortion, which can have significant effects on survivors [68,69].

Considering that sex offenders are regarded as one of the more serious offender groups [70,71], it is not surprising they were the second most commonly researched group in our sample (620/4814, 12.9%). European surveys have suggested that up to 10% of male offenders commit sexual violence against adult women [72,73], with Australia noting an average of 36.4% of all offenses recorded have been related to sexual assault in the last 15 years [74]. US statistics also put the prevalence of sexual assault at half a million incidents per year [75]. Despite male individuals comprising the majority of the sex offender population (eg, in Australia, 97% of sex offenders are men) [76], research suggests that the proportion of female sex offenders is higher than thought [77]. A recent meta-analysis with data from 12 countries reported that victimization surveys indicated that prevalence rates of female sex offenders were 6 times higher than official data (11.6%). This disparity is similar to our findings that saw female sex offenders comprising 13.1% (n=81) versus male sex offenders with 34% (n=211) from 620 abstract studies.

Despite an estimate of 1 billion children aged 12-17 years experiencing child abuse and maltreatment [78], our findings suggest that individuals responsible for committing such offenses are underresearched, with only 1.7% (84/4814) of the abstracts reporting child-related crimes. Since such offenses are hard to detect due to the involvement of minors and adolescents, with only 1.7% of our sample older than 70 years involving those convicted of child sex offenses, highlighting a research gap in justice health. To design and implement effective prevention and intervention programs for child-related crimes, it is necessary to conduct more evidence-based research on individuals committing this type of offense.

### Text Mining Error Analysis

#### Overview

Using a rule-based method returned encouraging results (the mean *F*_1_-score was 93% across the 4 characteristics), although the micro–*F*_1_-score was at 89.9%, which can offer a more weighted performance due to each class’s different number of mentions in the evaluation set. There was a higher number of FNs (36 in total) as opposed to FPs (13 in total), explaining the higher microprecision (94.3%) of our approach.

#### Generation of FPs

Although our method was effective in identifying the majority of nationality mentions of the participant population (90.9% precision with an increase of 4.5% from the development set), some nationality terms that were either part of a population’s ethnicity (eg, Mexican-American) or referred to nationalities irrelevant to the current study (eg, “As in the earlier (British) [FP] study” and “Despite being shown on alcohol-related harm as well as with young [FP] people in the USA”) led to the generation of a small number of FPs (4 in total). It is safe to assume that such cases can be present in our larger study sample despite their low prevalence as FPs in the evaluation set.

The use of generic terms (eg, “delinquent,” “criminal,” and “adolescent) to capture the age and offender type of populations also led to the generation of 1 FP (ie, “Maternal depression is a risk factor for adolescent [FP] depression”) and 8 FPs (eg, “that drug use was more strongly related to disruptive and delinquent [FP] behavior, for both sexes” and “drinking problems and criminal [FP] arrests were interrelated”), respectively. This indicates that perhaps more specific terms in our dictionaries could potentially limit the generation of FPs on that front. Nevertheless, both precision values were above 92% (97.6% for age; 92.4% for offender type), suggesting that their number of rules was enough to capture accurately this type of information.

Interestingly, there were no FPs for the characteristic of sex (100% precision), demonstrating that a simple rule-based approach relying on 4 rules to capture the participant’s sex from PubMed justice health abstracts can produce reliable results.

#### Generation of FNs

The lack of implemented rules due to not being previously observed in our training and development sets’ syntactical patterns was as a source of FNs, particularly in age (eg, “majority of these incarcerated youth [FN] have one” and “considerations for minors [FN] facing delinquency”), sex (eg, “Males [TP] greatly outnumbered females [FN]” and “males [TP] were less likely than females [FN] to have”), and offender type (eg, “which girls committed aggressive offenses [FN]” and “residential location of a serial offender [FN]”).

Another source of errors was the presence of misspellings in the published text that did not trigger the respective rules for age (eg, “compared between 12 and 14-year-old [FN] boys who attended a delinquency”), resulting in age having the lowest recall of all 4 characteristics (78.8%). The lack of terms from our offender-type dictionaries (eg, “individuals who are subject to a restraining order or have been convicted of a domestic violence misdemeanor [FN]” and “serial commercial robber”) also generated FNs and, in combination with unencountered syntactical patterns that were not implemented in our rules, resulted in the second lowest recall (83.7%; 19 FNs). This emphasizes the necessity for expanding the rule and dictionary scope to capture several other descriptions of this characteristic in the published abstract text.

Although 2 nationality mentions (eg, “offenders committed to Iowa Department of Corrections [FN]” and “records of three private Minnesota [FN] adoption agencies”) were incorrectly ignored by our approach, the recall was the highest from all characteristics with 95.2% suggesting that the existing coverage of our rules was sufficient.

### Limitations

Our study comes with several limitations. First, PubMed articles might not be sufficient to portray a complete picture of offending and incarcerated populations since government articles and reports can remain unpublished and so fall outside the scope of this study. Second, research with a sociological and criminological focus is unlikely to appear in journals covered by PubMed. Thus, our dataset could potentially underestimate the total number of research outputs in this area. Third, we focused only on English-language abstracts, which carried a risk of “English-language” bias. However, the incorporation of non–English-language abstracts in our PubMed sample could ensure greater research transparency and findings and reduce bias.

Fourth, using only abstract text almost certainly does not give a full picture with regard to the investigated population. As noted in our findings, only a fraction of abstracts reported any of the 4 characteristics we examined: offender type (4814/34,481, 13.9%), nationality (9525/34,481, 27.6%), age (5170/34,481, 14.9%), and sex (8169/34,481, 23.7%). It is likely that full-text articles, especially those that might adhere to official reporting guidelines (eg, PRISMA and STROBE), detail their population reporting in the body text of the article, which would elicit different findings than those presented here. However, this was not feasible and would have involved permissions from numerous publishing houses and be extremely costly. In addition, changes over time in local and global research priorities including publication practices are likely to have influenced the results. Thus, our result interpretation should be taken with caution and mindfully of these macro-level influences.

Finally, despite a reliable performance from our methodology, the number of identified characteristics could be underrepresented (especially for age and offense type). Using specific rules might not have been enough to identify all mentions of age while more descriptive cases for offender types could have resulted in FNs. It is possible that a hybrid approach that uses both machine learning and rules will limit the number of FNs and thus enhance the accuracy of identifying these types of population characteristic mentions.

### Conclusions

Our study demonstrated that it is feasible and efficient to extract key information from populations within a large sample of justice health study abstracts over time. Our findings align with existing research that has highlighted a focus on female offender studies and has revealed an emphasis on offenders with mental illness and minors with rising rates in the last 30 years. Interestingly, research involving child crime–related offenders was not common. Despite the United States having the largest incarcerated population in the world, adjusting its publication rate by the prisoner population demonstrates that Nordic countries with progressive approaches to offender rehabilitation have published proportionately more research. Our findings offer new insights into the whole area of justice health, with clear implications to promote diversity in cohort selection and limitation of bias and research gaps.

## Data Availability

The datasets generated or analyzed during this study are available from the corresponding author on reasonable request. No generative artificial intelligence was used in any part of the manuscript.

## Appendix

Multimedia Appendix 1 Terms used to identify the population's sex in PubMed abstracts.

Multimedia Appendix 2 Terms used to describe offending and incarcerated populations in PubMed abstracts.

Multimedia Appendix 3 Rule examples for each population characteristic (ie, age, sex, nationality, and offender type).

Multimedia Appendix 4 Miscellaneous terms used to describe offending and incarcerated populations in PubMed abstracts.

Multimedia Appendix 5 Number of published articles (n=34,481) in PubMed related to epidemiological criminology from 1963 to 2023.

Multimedia Appendix 6 Rates of justice health abstracts (n=8169) in PubMed that reported male-only, female-only, transgender-only, and both female and male populations from 1990 to 2023.

Multimedia Appendix 7 Number of justice health abstracts (n=4814) in PubMed that mention one offender characteristic in 1 of the 6 defined group classes.

Multimedia Appendix 8 Mental health concepts related to offending populations in justice health PubMed abstracts.

## Abbreviations

CONSORT: Consolidated Standards of Reporting Trials
FN: false negative
FP: false positive
GATE: General Architecture for Text Engineering
PRISMA: Preferred Reporting Items for Systematic Reviews and Meta-Analyses
SPIRIT: Standard Protocol Items: Recommendations for Interventional Trials
STROBE: Strengthening the Reporting of Observational Studies in Epidemiology
TP: true positive
