# Supplementary material for: Population Characteristics in Justice Health Research Based on PubMed Abstracts From 1963 to 2023: Text Mining Study
Source: JMIR Form Res. 2024 Nov 22;8:e60878. doi: 10.2196/60878 (PMC11624456; doi:10.2196/60878)
Supplement: Multimedia Appendix 1 [file formative_v8i1e60878_app1.docx]

Terms used to identify the population’s sex in PubMed abstracts.

| Male | Men | Boy | Transexual | Trans | Transfemale |
| --- | --- | --- | --- | --- | --- |
| Female | Women | Girl | Transsexual | FTM | Transfeminine |
| Males | Mothers | Boys | Transgendered | MTF | Transmale |
| Females | Fathers | Girls | Transgender | Transmale | Transmasculine |
| Transfeminine | Intersex |  | | | |
