# Supplementary material for: Population Characteristics in Justice Health Research Based on PubMed Abstracts From 1963 to 2023: Text Mining Study
Source: JMIR Form Res. 2024 Nov 22;8:e60878. doi: 10.2196/60878 (PMC11624456; doi:10.2196/60878)
Supplement: Multimedia Appendix 2 [file formative_v8i1e60878_app2.docx]

Terms used to describe offending and incarcerated populations in PubMed abstracts.

| Aboriginal | Contact | Gender binary | Intergender | Mildly violent | Psychiatrically evaluated | Transmasculine |
| --- | --- | --- | --- | --- | --- | --- |
| Adjudicated | Convicted | Gender dysphoria | Intersex | Misdemeanant | Psychiatrically examined | Transsexual |
| Admitted | Criminal | Gender expansive | Intersexual | Misgender | Psychiatrically hospitalized | Treated |
| Adolescent | Dangerous | Gender expression | Intrafamilial | Moderate risk | Psychopathic | Trigender |
| Adult | Daughter | Gender fluid | Juvenile | Moderately aggressive | Psychotic | Two spirit |
| Afab | Deemed not guilty by reason of insanity | Gender identity | Killer | Moderately violent | Queer | Two spirited |
| Agender | Delinquent | Gender nonconforming | Late | Moderate-risk | Remand | Twospirit |
| Aggressive | Demiboy | Gender normative | Late adolescent | Mother | Reoffending | Two-spirit |
| Aliagender | Demigender | Gender presentation | Late first | Mtf | Re-offending | Two-spirited |
| Amab | Demigirl | Gender queer | Late starter | Multi-gender | Repeated | Very high risk |
| Androgyne | Dependent | Gender questioning | Late-adolescent | Native | Reported | Very high-risk |
| anti social | Detained | Gender roles | LBT | Neutrois | Schizophrenic | Victimized |
| Antisocial | Detained | Gender variant | Legally involved | Ngri | Second time | Victimized |
| Anti-social | Drug | Genderfluid | Lesbian | Non maltreated | Second-time | Violent |
| Antisocial personality disordered | Drug involved | Genderfuck | LGBT | Non psychotic | Self reported | Women |
| Aporagender | Drug-involved | Gender-neutral | LGBTIQ | Non schizophrenic | Self-reported | Young |
| Apprehended | Dyadic | Gender-neutral pronouns | LGBTIQ+ | Nonbinary | Serial | Younger |
| Arrested | Early | Genderqueer | LGBTQ | Non-binary | Serious | Youth |
| Asexual | Early adolescent | Gendervoid | LGBTQI | Noncontact | Severely aggressive | Youthful |
| Assigned female at birth | Early first | Girl | Lifelong | Nonconvicted | Severely violent |  |
| Assigned male at birth | Early starter | Graygender | Low iq | Nonmaltreated | Sex |  |
| Bigender | Early-adolescent | Habitually | Low risk | Nonpsychopathic | Sex assigned at birth |  |
| Binarism | Ecstasy | Heroin | Low-iq | Non-psychotic | Sexual |  |
| Bisexual | Extrafamilial | Heterosexual | Low-risk | Non-schizophrenic | Sexually |  |
| Body dysphoria | Father | High- and low-risk | Lsd | Nonsex | Social dysphoria |  |
| Boi | Female | High iq | Male | Novigender | Sociopathic |  |
| Boy | Female-to-male | High risk | Male-to-female | Offending | Soft butch |  |
| Butch | Feminine of center | High-iq | Maltreated | Omnigender | Son |  |
| Charged | Feminine presenting | High-risk | Marijuana | Opium | Stone butch |  |
| Child | Feminine-of-center | Homeless | Masculine of center | Outpatient | Straight |  |
| Child sexual | Feminine-presenting | Homosexual | Masculine presenting | Pangender | Substance |  |
| Chronic | Femme | Ice | Masculine-of-center | Pansexual | Suburban |  |
| Cisgender | First time | Impoverished | Masculine-presenting | Partnered | Third gender |  |
| Cishet | First-time | Incarcerated | Maverique | Peer | Trans feminine |  |
| Cisnormativity | Fist time | Indigenous | Men | pilot polygraph | Trans masculine |  |
| Cissexism | Found not guilty by reason of insanity | Inhalant | Mental ill | Polygender | Transexual |  |
| Clinical | Ftm | Injecting drug | Mentally disordered | Pre pubescent | Transfeminine |  |
| Cocaine | FZ | Imprisoned | Mentally ill | Pre-pubescent | Transgender |  |
| Community supervised | Gay | Institutionalized | Meth | Prison | Transgendered |  |
| Community-supervised | Gender apathetic | Institutionalized | Mildly aggressive | Prosecuted | Transitioning |  |
