# Supplementary material for: Population Characteristics in Justice Health Research Based on PubMed Abstracts From 1963 to 2023: Text Mining Study
Source: JMIR Form Res. 2024 Nov 22;8:e60878. doi: 10.2196/60878 (PMC11624456; doi:10.2196/60878)
Supplement: Multimedia Appendix 3 [file formative_v8i1e60878_app3.docx]

Rule examples for each population (i.e., age, sex, nationality, offender type) characteristic. The text in bold is the information identified by the rules.

| **Offender type** |  |  |  |  |  |  |
| --- | --- | --- | --- | --- | --- | --- |
| **Example** | sample | of |  | **incarcerated** |  | **offenders** |
| **Rule** | {Token.string==~"(?i)sample\|majority"} | {Token.string==~"(?i)of"} | ({Token})[0,1] | ({Lookup.majorType=="descriptive"})? | ({Lookup.majorType=="nationality"})? | ((type)\|  {Lookup.majorType=="offenders"}\|  (young)\|  {Token.string==~"(?i)youth"}) |
|  | | | | | | |
| **Nationality** |  |  |  |  |  |  |
| **Example** | in | the |  | **United Kingdom** |  |  |
| **Rule** | {Token.string==~"(?i)in\|inside\|among"} | {Token.string==~"(?i)the"} | ({Token})[0,1] | ({Lookup.majorType=="states"}\|  {Lookup.majorType=="uscities"}\|  {Lookup.majorType=="cities"}\|  {Lookup.majorType=="country"}\|  {Lookup.majorType=="province"}\|{Lookup.majorType=="counties"}) |  |  |
|  | | | | | | |
| **Sex** |  |  |  |  |  |  |
| **Example** | in | 194 | **male** | inmates |  |  |
| **Rule** | - | - | ({Token.string==~"(?i)male\|female\|males\|females\|men\|women\|mothers\|fathers\|boy\|girl\|boys\|girls"}) | - |  |  |
|  | | | | | | |
| **Age** |  |  |  |  |  |  |
| **Example** | among | male | **adolescent** |  | offenders |  |
| **Rule** | {Token.string==~"(?i)among\|for\|from"} | ({Token.string==~"(?i)male\|female"})? ({Token.string==~"(?i)male\|female"})? | ({Token.string==~"(?i)juvenile\|young\|youth\|adolescent\|minor\|teenager\|delinquent\|teen"} | ({Token.string==~"(?i)adult"})?) | {Token.string==~"(?i)offenders\|detainees\|delinquents\|girls\|boys\|remandees\|prisoners\|criminals\|perpetrators"} |  |
