# Supplementary material for: Population Characteristics in Justice Health Research Based on PubMed Abstracts From 1963 to 2023: Text Mining Study
Source: JMIR Form Res. 2024 Nov 22;8:e60878. doi: 10.2196/60878 (PMC11624456; doi:10.2196/60878)
Supplement: Multimedia Appendix 4 [file formative_v8i1e60878_app4.docx]

Miscellaneous terms used to describe offending and incarcerated populations in PubMed abstracts.

| Children | Indigenous | Adult | Neurological condition | Adjudicated | First time | Ex-offender |
| --- | --- | --- | --- | --- | --- | --- |
| Male | Female | Juvenile | High risk | Gang member | Traumatic brain injury | Disease |
| Adolescent | Delinquent | Young | Minor | Early starter | Serial | Aggressive behavior |
| Homosexual | Injury | Homeless | First degree | Minors | Intrafamilial | Migrain |
| Heterosexual | Mother | Ex-offenders | Father | Non-psychotic disorder | Late onset | Justice system |
| Adolescents | NGRI | Online | LGBT | Father absent | Extrafamilial | asthma |
| Irritable bowel syndrome | Promoter | Sex reassigned | Late start | Sadomasochism | Cancer |  |
