# Supplementary figures and images for: Population Characteristics in Justice Health Research Based on PubMed Abstracts From 1963 to 2023: Text Mining Study
Source: JMIR Form Res. 2024 Nov 22;8:e60878. doi: 10.2196/60878 (PMC11624456; doi:10.2196/60878)

Number of published articles (*n* = 34,481) in PubMed related to epidemiological criminology from 1963 to 2023.


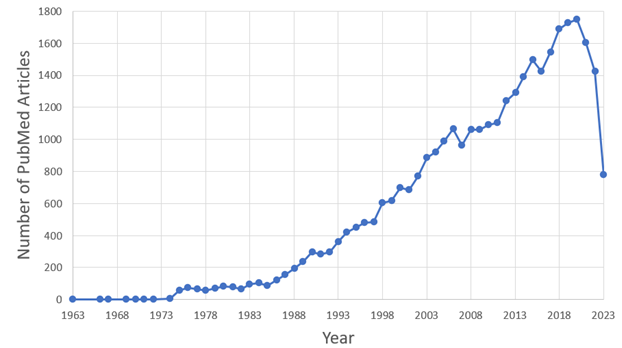

Supplement: Multimedia Appendix 5 [file formative_v8i1e60878_app5.docx]
