# Supplementary material for: Population Characteristics in Justice Health Research Based on PubMed Abstracts From 1963 to 2023: Text Mining Study
Source: JMIR Form Res. 2024 Nov 22;8:e60878. doi: 10.2196/60878 (PMC11624456; doi:10.2196/60878)
Supplement: Multimedia Appendix 6 [file formative_v8i1e60878_app6.docx]

Rates of justice health abstracts (n=8,169) in PubMed that reported male only, female only, trans only and both female and male populations from 1990 to 2023.

| **Year** | **Male rate** | **Female rate** | **Transgender** | **Both female and male** |
| --- | --- | --- | --- | --- |
| 1990 | 50.8 | 29.2 | 0.0 | 20.0 |
| 1991 | 34.5 | 34.5 | 0.0 | 30.9 |
| 1992 | 44.9 | 38.8 | 0.0 | 16.3 |
| 1993 | 33.3 | 46.2 | 0.0 | 20.5 |
| 1994 | 30.9 | 40.7 | 0.0 | 28.4 |
| 1995 | 42.3 | 44.2 | 0.0 | 13.5 |
| 1996 | 40.9 | 42.7 | 0.0 | 16.4 |
| 1997 | 34.7 | 42.4 | 0.0 | 22.9 |
| 1998 | 40.9 | 41.6 | 0.0 | 17.5 |
| 1999 | 43.8 | 35.2 | 0.0 | 21.1 |
| 2000 | 33.1 | 43.3 | 0.6 | 22.9 |
| 2001 | 35.8 | 42.5 | 0.0 | 21.6 |
| 2002 | 39.4 | 40.4 | 0.0 | 20.2 |
| 2003 | 45.2 | 38.1 | 0.0 | 16.8 |
| 2004 | 41.6 | 40.1 | 0.0 | 18.3 |
| 2005 | 40.2 | 39.7 | 0.0 | 20.1 |
| 2006 | 41.7 | 39.1 | 0.0 | 19.1 |
| 2007 | 40.5 | 39.6 | 0.0 | 19.8 |
| 2008 | 44.6 | 36.4 | 0.0 | 19.0 |
| 2009 | 38.1 | 39.3 | 0.0 | 22.6 |
| 2010 | 40.2 | 39.8 | 0.0 | 19.9 |
| 2011 | 42.3 | 40.4 | 0.0 | 17.2 |
| 2012 | 42.2 | 41.6 | 0.0 | 16.2 |
| 2013 | 42.6 | 36.9 | 0.3 | 20.2 |
| 2014 | 38.6 | 45.8 | 0.0 | 15.6 |
| 2015 | 39.7 | 40.8 | 0.0 | 19.4 |
| 2016 | 36.5 | 43.8 | 0.0 | 19.7 |
| 2017 | 38.0 | 46.1 | 0.3 | 15.7 |
| 2018 | 42.6 | 42.6 | 0.3 | 14.6 |
| 2019 | 38.2 | 48.0 | 0.0 | 13.8 |
| 2020 | 36.8 | 47.8 | 0.2 | 15.2 |
| 2021 | 34.8 | 53.9 | 0.2 | 11.1 |
| 2022 | 35.1 | 54.2 | 0.3 | 10.4 |
| 2023 | 33.3 | 48.6 | 0.9 | 17.1 |
