# Supplementary material for: Population Characteristics in Justice Health Research Based on PubMed Abstracts From 1963 to 2023: Text Mining Study
Source: JMIR Form Res. 2024 Nov 22;8:e60878. doi: 10.2196/60878 (PMC11624456; doi:10.2196/60878)
Supplement: Multimedia Appendix 7 [file formative_v8i1e60878_app7.docx]

Number of justice health abstracts (n=4,814) in PubMed that mention one offender characteristic in one of the six defined group classes. Note that one abstract might have more than one different characteristic mentions.

| **Year** | **Child crime related offenders** | **Non-violent offenders** | **Sex offenders** | **Mentally ill offenders** | **Drug related offenders** | **Violent offenders** |
| --- | --- | --- | --- | --- | --- | --- |
| 1990 | 1 | 2 | 9 | 7 | 7 | 4 |
| 1991 | 1 | 0 | 4 | 4 | 4 | 2 |
| 1992 | 0 | 0 | 3 | 7 | 4 | 6 |
| 1993 | 1 | 0 | 6 | 5 | 8 | 4 |
| 1994 | 5 | 1 | 12 | 9 | 7 | 4 |
| 1995 | 0 | 2 | 8 | 13 | 6 | 3 |
| 1996 | 1 | 0 | 3 | 9 | 4 | 6 |
| 1997 | 1 | 0 | 7 | 13 | 5 | 10 |
| 1998 | 1 | 4 | 12 | 17 | 14 | 9 |
| 1999 | 0 | 2 | 10 | 21 | 11 | 5 |
| 2000 | 2 | 3 | 13 | 17 | 13 | 5 |
| 2001 | 1 | 5 | 13 | 17 | 15 | 11 |
| 2002 | 5 | 4 | 23 | 25 | 16 | 8 |
| 2003 | 3 | 7 | 25 | 18 | 18 | 12 |
| 2004 | 6 | 2 | 35 | 26 | 17 | 18 |
| 2005 | 1 | 4 | 21 | 18 | 17 | 11 |
| 2006 | 3 | 4 | 29 | 24 | 20 | 13 |
| 2007 | 2 | 4 | 27 | 34 | 25 | 13 |
| 2008 | 5 | 4 | 33 | 44 | 27 | 15 |
| 2009 | 6 | 3 | 29 | 28 | 25 | 17 |
| 2010 | 2 | 4 | 26 | 38 | 17 | 9 |
| 2011 | 4 | 4 | 20 | 25 | 20 | 11 |
| 2012 | 8 | 3 | 34 | 32 | 18 | 9 |
| 2013 | 6 | 4 | 34 | 46 | 17 | 16 |
| 2014 | 1 | 6 | 14 | 36 | 22 | 15 |
| 2015 | 2 | 4 | 17 | 33 | 23 | 10 |
| 2016 | 1 | 1 | 20 | 29 | 21 | 7 |
| 2017 | 1 | 1 | 17 | 34 | 15 | 18 |
| 2018 | 6 | 5 | 22 | 38 | 18 | 17 |
| 2019 | 4 | 3 | 18 | 36 | 14 | 19 |
| 2020 | 0 | 2 | 22 | 40 | 14 | 10 |
| 2021 | 0 | 2 | 10 | 19 | 14 | 8 |
| 2022 | 0 | 2 | 9 | 29 | 7 | 10 |
| 2023 | 1 | 0 | 8 | 14 | 9 | 4 |
