# Supplementary material for: Population Characteristics in Justice Health Research Based on PubMed Abstracts From 1963 to 2023: Text Mining Study
Source: JMIR Form Res. 2024 Nov 22;8:e60878. doi: 10.2196/60878 (PMC11624456; doi:10.2196/60878)
Supplement: Multimedia Appendix 8 [file formative_v8i1e60878_app8.docx]

Mental health concepts related to offending populations in justice health PubMed abstracts.

| Mental illness | Inhalant abuse | Obsessive compulsive disorder |
| --- | --- | --- |
| Substance use disorder | Specific reading retardation | Developmental disorder |
| Intellectual disability | Insanity | Selective mutism |
| Neurodevelopmental disorder | Dyslexia | Developmental delay |
| Schizophrenia | Schizoaffective disorder | Dissociative identity disorder |
| Psychopath | Eating disorder | Pervasive developmental disorder |
| Adhd | Schizophrenia spectrum psychosis | Self harm |
| Alcoholic | Language delay | Anxiety |
| Antisocial personality disorder | Developmental coordination disorder | Down syndrome |
| Dementia | Suicide attempt | Psychotic symptoms |
| Psychosis | Rett syndrome | Alzheimer’s |
| Autism | Anorexia nervosa | Behavior disorder |
| Personality disorder | Depressive symptoms | Mood disorder |
| Heroin abuse | Delusional disorder | Suicidal ideation |
| Depression | Tourette | Opioid abuse |
| Borderline personality disorder | Chemical abuse | Pedophilia |
| Learning disability | Generalized anxiety disorder | Hare psychopathy |
| Conduct disorder | Oppositional defiance disorder | Disruptive behavior disorder |
| Bipolar | Mental retardation | Cocaine abuse |
| Ptsd |  |  |
